# Machine Learning and Lean Six Sigma to Assess How COVID-19 Has Changed the Patient Management of the Complex Operative Unit of Neurology and Stroke Unit: A Single Center Study

**DOI:** 10.3390/ijerph19095215

**Published:** 2022-04-26

**Authors:** Giovanni Improta, Anna Borrelli, Maria Triassi

**Affiliations:** 1 Department of Public Health, University of Naples “Federico II”, 80131 Naples, Italy; triassi@unina.it; 2 Interdepartmental Center for Research in Healthcare Management and Innovation in Healthcare (CIRMIS), University of Naples “Federico II”, 80131 Naples, Italy; 3 “San Giovanni di Dio e Ruggi d’Aragona” University Hospital, 84121 Salerno, Italy; acquarama@libero.it

**Keywords:** Six Sigma, health care, DMAIC, clinical pathway, COVID-19, statistical analysis

## Abstract

**Background:** In health, it is important to promote the effectiveness, efficiency and adequacy of the services provided; these concepts become even more important in the era of the COVID-19 pandemic, where efforts to manage the disease have absorbed all hospital resources. The COVID-19 emergency led to a profound restructuring—in a very short time—of the Italian hospital system. Some factors that impose higher costs on hospitals are inappropriate hospitalization and length of stay (LOS). The length of stay (LOS) is a very useful parameter for the management of services within the hospital and is an index evaluated for the management of costs. **Methods:** This study analyzed how COVID-19 changed the activity of the Complex Operative Unit (COU) of the Neurology and Stroke Unit of the San Giovanni di Dio e Ruggi d’Aragona University Hospital of Salerno (Italy). The methodology used in this study was Lean Six Sigma. Problem solving in Lean Six Sigma is the DMAIC roadmap, characterized by five operational phases. To add even more value to the processing, a single clinical case, represented by stroke patients, was investigated to verify the specific impact of the pandemic. **Results:** The results obtained show a reduction in LOS for stroke patients and an increase in the value of the diagnosis related group relative weight. **Conclusions:** This work has shown how, thanks to the implementation of protocols for the management of the COU of the Neurology and Stroke Unit, the work of doctors has improved, and this is evident from the values of the parameters taken into consideration.

## 1. Introduction

The pandemic caused by coronavirus disease 2019 (COVID-19) has radically changed the medical landscape in every aspect [1]. The infection with SARS-CoV-2 mainly affects the respiratory system and lungs, but the virus has also been shown to affect other compartments of the body such as the eyes, heart, skin, kidneys and central nervous system (CNS) [2]. Several studies have shown how patients with COVID-19 showed the presence of neurological manifestations [3,4,5]. During this period, neurologists were involved on the front line and found themselves watching over the neurological complications of COVID-19. In fact, patients with neurological disorders, particularly those on immunomodulatory therapy, will require careful monitoring [6]. Although the goal is to put in place protective measures for patients hospitalized due to COVID-19 infection, other difficulties may also arise, such as the availability of side rooms and the logistics of the social distancing of patients, which has been shown to minimize spread [7].

Therefore, all the measures adopted by the government, such as the lock-down, social distancing, the blocking of deferred elective procedures and the management of the pandemic, have inevitably changed the normal activity of the hospital departments, including the neurology department.

The optimization of care processes with a view toward global improvement and the containment of expenditure become elements of fundamental importance in the whole context analyzed. By means of performance-oriented techniques and approaches, borrowed from the manufacturing industry [8,9,10,11] and applied to the healthcare sector [12,13,14,15,16], such as Lean Six Sigma (LSS) [17,18,19,20,21,22], technology assessment [23,24,25,26,27] and big data analysis through machine learning and deep learning algorithms [28,29,30], promising results have been achieved in terms of the improvement in the quality and efficiency of healthcare services, and new methodologies and technologies have been proposed to improve diagnostic [31,32,33,34] or therapeutic pathways and procedures [35,36,37,38,39]. Among the novel care paradigms, telemedicine and telemonitoring, with the provision of remote services [40], have contributed to obtaining benefits for the management of patients, especially those with movement disorders, who are most affected by the effects of the lock-down [41].

The critical situation due to the COVID-19 pandemic has contributed to obtaining benefits regarding inappropriate hospitalizations. Factors that are associated with inappropriate hospitalizations have been shown to be the patient’s age, functional and health status and specialty of care [42,43].

In this study, we intend to evaluate how the influence of the COVID-19 pandemic has influenced the activity of the Complex Operative Unit (COU) of the Neurology and Stroke Unit of the San Giovanni di Dio and Ruggi d’Aragona University Hospital. In addition, we also focus on a specific category of patients, i.e., stroke patients, in order to evaluate the impact of the pandemic on a clinical case. In fact, stroke is the most common reason for hospitalization in the processed dataset, with a percentage that exceeds 30% of the total number of patients treated. Although several analyses have been made in recent years to prevent and manage infections and disease [44,45,46,47], and, within the last two years, to analyze, understand and predict the dynamics and evolution of the COVID-19 pandemic [48,49,50], the majority of this research has focused on how the virus spreads and what factors most impact this spread. In the proposed analysis, we are focusing on the patients in order to evaluate and improve the understanding of the COVID-19 pandemic on a cohort of 1538 subjects. In particular, this study is an extension of a previous work in which the impact of COVID-19 was analyzed on a limited number of cases (hospitalized patients) using statistical analysis and regression models, 845 patients of which were admitted in 2019 and 693 in 2020 [51]. A Six Sigma (SS) methodology was applied in the study. In previous years, many researchers have applied an SS methodology to analyze clinical pathways in different fields of medicine [52,53,54]. Apart from medical procedures, LSS also facilitates administrative management, including medical recordkeeping, finance management, patient hospitalization and discharge forms and medical equipment coding [52]. In this work, we will analyze changes in terms of length of hospital stay (LOS), mode of discharge and diagnosis related group (DRG) relative weight.

## 2. Materials and Methods

**Figure 1:**
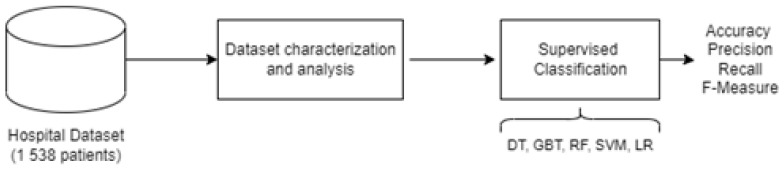
Overall flow of the proposed analysis from the patient’s point of view based on machine learning models.

In this analysis, we propose a machine learning-based methodology, whose aim is to investigate the effect of the COVID-19 pandemic on a large cohort of patients. In particular, Figure 1 summarizes the overall flow of our analysis.

We first collected different data about a cohort of 1583 patients from 2019–2021. In particular, this study was conducted at the COU of the Neurology and Stroke Unit of the San Giovanni di Dio e Ruggi d’Aragona University Hospital in Salerno (Italy). All patients admitted in the years 2018–2019 (before COVID-19) and 2020–2021 (during COVID-19) were considered in the study. The dataset was extracted from the hospital’s information system, the QuaniSDO. The extracted information was:

- Gender,
- Age,
- Department and COU,
- Main and secondary diagnoses,
- Diagnosis related group (DRG),
- Length of hospital stay (LOS), i.e. date of admission—date of discharge,
- Diagnosis related group (DRG) relative weight,
- Number of days of hospital (DH) admission, i.e. the number of visits to the hospital for appointments and medical checks,
- Mode of discharge.

Using the DRG, it was possible to identify patient discharge pathologies. From this information, a subgroup was extracted from the dataset, i.e., stroke patients, in order to assess the impact of COVID-19 on this specific class of patient. By analyzing the main and secondary diagnoses, it was possible to identify the main comorbidities (hypertension, atrial fibrillation and atherosclerosis) of stroke patients, which were then used as input variables to the ML algorithms. Finally, different machine learning models were used for classifying patients between the two identified classes.

### 2.1. Data Analysis

Microsoft Excel (version 2016) and IBM SPSS (Statistical Package for Social Science) Statistics (version 27) were used to study the dataset. In particular, a Pareto diagram was created using MS Excel v2016 Software, while statistical analysis was implemented with the support of IBM SPSS Statistics v27 Software. First, the distribution was tested using the Kolmogorov–Smirnov test, with a significance level of 95%. For each variable, the distribution normality was not demonstrated. For this reason, the Mann–Whitney U test (MW), the Kruskal–Wallis test and the chi-squared test, with a 95% confidence interval, were used. For the dataset consisting of stroke patients only, after performing the comparative statistical analysis, 5 ML algorithms—decision trees (DT), random forest (RF), support vector machine (SVM), logistic regression (LR) and gradient boosted trees (GBT)—were implemented to verify the possibility of classifying patients between the two identified classes. To do this, a Python script was created.

#### Machine Learning Algorithms

The use of machine learning models to support supervised classification tasks has been applied, achieving high efficacy performances in different domains (i.e., finance [55], security [56], music [57], and healthcare [58,59,60,61,62,63]). In particular, these methods mainly rely on two phases: feature selection, that has been discussed in Section 2.1, and classification.

Concerning the second phase of the proposed methodology, we evaluate the efficacy performances of several well-known machine learning algorithms. In particular, DT is a simple classification model based on the construction of decision trees. At the level of each node, a feature of the variables is checked. The result of this comparison determines the choice of a specific branch to get to the next node. The cost of using the tree (i.e., predicting data) is logarithmic in the number of data points used to train the tree, but small variations in the data might result in a generation of completely different trees. GBT and RF use the DT model and strengthen it through the progressive combination of weak predictors for performance improvement. SVM, however, uses a hyperplane in an N-dimensional space (N—the number of features) defined through the use of a loss function in order to maximize the distance between the points of different classes. Lastly, LR is a linear model for classification, where the probabilities describing the possible outcomes of a single trial are modeled using a logistic function.

For all algorithms, the dataset was divided into a training set and a test set, respectively, at 80% and 20%, and an initial oversampling was performed in order to increase the size of the rare samples (Class 2).

### 2.2. DMAIC Cycle

In accordance with the problem, solving solution provided by the methodology, the project was divided into five phases, each coinciding with one of the DMAIC roadmap steps [53]. The simplified procedures of each phase are [54]:

- Define by identifying, prioritizing and selecting the correct project;
- Measure key process characteristic, the scope of parameters and their performances;
- Analyze by identifying key causes and process determinants;
- Improve by changing the process and optimizing performance; and
- Control by sustaining the gain.

In this framework, both soft Lean and Six Sigma tools have been combined. In addition, the use of additional predictive tools for data analysis and measurements, namely regression and machine learning classifiers, strengthen the Six Sigma approach compared to the Lean method.

#### 2.2.1. Define

During the “define” phase, the purpose of the work and the improvements to be implemented to the process are identified. The identified CTQ (critical to quality) measurement was the LOS, the mode of discharge and DRG relative weight.

The LOS—measured in days—is defined as the difference between the date of admission and the date of discharge of the patient.

All the aspects of the project were clarified in a project chart (Table 1).

SIPOC analysis has been performed in this “define” phase; this diagram is generally used to identify all relevant elements of a process improvement project before work begins. SIPOC stands for supplier, input, process, output and customer (Table 2).

#### 2.2.2. Measure

In this phase, measures of the process before the improvement were carried out. First, we collected data from 1 January 2018 to 31 December 2019 concerning all admissions to the Neurological Clinic and Stroke Unit, representing the pre-pandemic data. The second dataset was collected in the year 2020 and 2021, representing the period affected by the pandemic of COVID-19. The information in Table 3 was collected for all patients: gender, age, mode of discharge, DRG relative weight, number of DH admissions and LOS.

#### 2.2.3. Analyze

**Figure 2:**
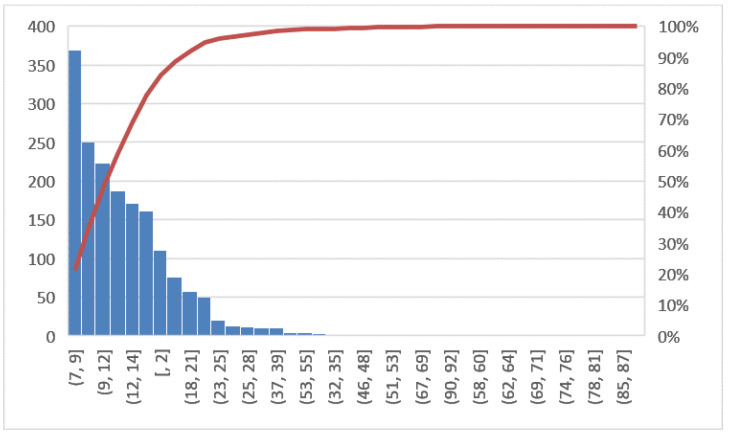
Pareto diagram of LOS (2018, 2019).

**Figure 3:**
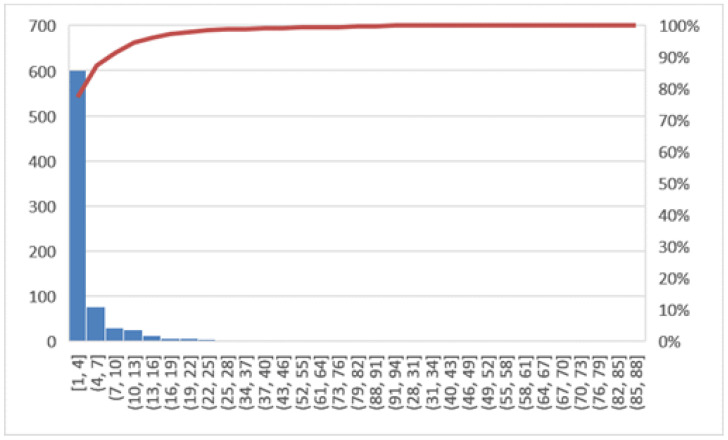
Pareto diagram of number of hospital admissions (2018, 2019).

The objectives of this phase will be to verify if the potential causes, previously identified as those that triggered the problem under consideration, are actually the correct reasons and to have the support of the confirmation derived from the analysis of the data. The tool used in this phase is the histogram. In Figure 2, the line graph shows the LOS trend in the years 2018 and 2019 for all hospitalized patients, while Figure 3 shows the Pareto diagram of the number of hospital admissions for patients with day hospital admissions in the two years 2018–2019.

**Figure 4:**
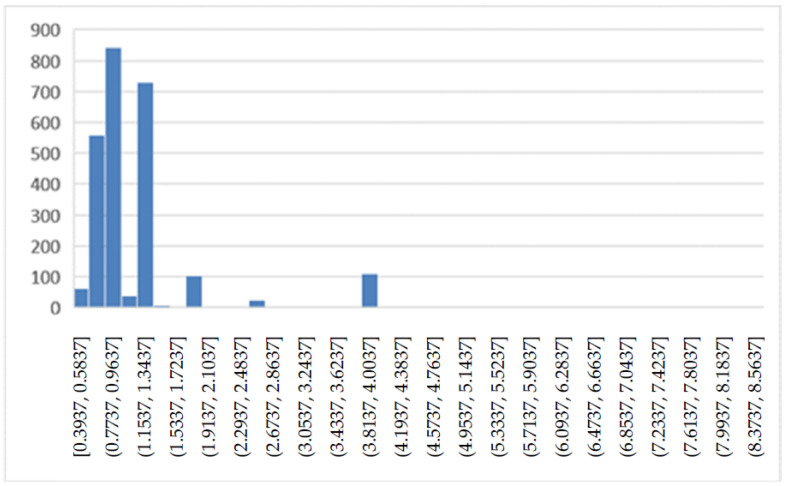
DRG relative weight (2018, 2019).

Figure 4 shows the trend of the DRG relative weight in the years 2018 and 2019. The histogram shows how often the values occur within the dataset.

**Figure 5:**
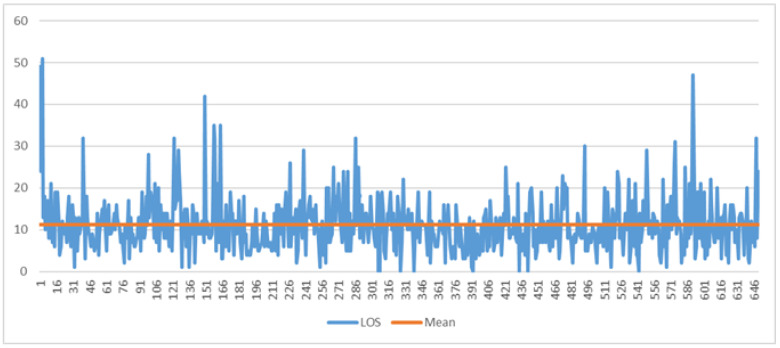
The run chart of total LOS for stroke patients before COVID-19. The orange line indicates the average value of 11.19 days.

Lastly, Figure 5 reports the change in LOS in days for all 650 observations relating to stroke patients in the 2 years 2018–2019. The orange line shows the average value of 11.19 days.

#### 2.2.4. Improve

Only after having collected and examined all the objective evidence is it possible to move on to the actual improvement phase. The purpose of this phase is to design the most suitable solution to solve the problem we are considering.

The COVID-19 emergency, with the necessary social distancing measures and the inevitable fear of the population to go to the hospital or contact local emergency services, has led to a significant decrease in the number of hospitalizations for stroke and therefore, in the number of patients treated compared to the numbers from the same period in the previous year.

The COVID-19 emergency has suddenly changed the geography of the stroke units: stroke is a health emergency but, even during the pandemic, high-level treatment was guaranteed.

The Campania region of Italy has reorganized the stroke network based on the number of hospitalizations for stroke by province and the respective population density, establishing three second-level stroke units and four first-level stroke units for the province of Naples and one second-level stroke unit for each of the remaining provinces. An additional first-level stroke unit, based on population density, has also been established [64].

The challenges and limitations faced in the management of patients of the COU of the Neurology and Stroke Unit induce medical specialists to come up with alternative solutions (Table 4).

#### 2.2.5. Control

The control phase is the final stage where monitoring tools are used to monitor the process.

In this phase, the real effects of the protocols adopted during the COVID-19 pandemic in the COU of the Neurology and Stroke Unit are assessed.

To evaluate the effects and show the differences in the parameters in the two years considered for the analysis, the tool used in this phase is also the histogram. In addition, to support the analysis, logistic regression and descriptive statistic methods were also applied. The results are shown in the next section.

## 3. Results

We organized our results into two main sections. First, we compared the results between before and after the COVID-19 emergency on the basis of data from the Neurology and Stroke Unit of the San Giovanni di Dio e Ruggi d’Aragona University Hospital in Salerno (Italy). Successively, different machine learning models were evaluated for classifying patients between the two identified classes.

### 3.1. Statistical Analysis

**Figure 6:**
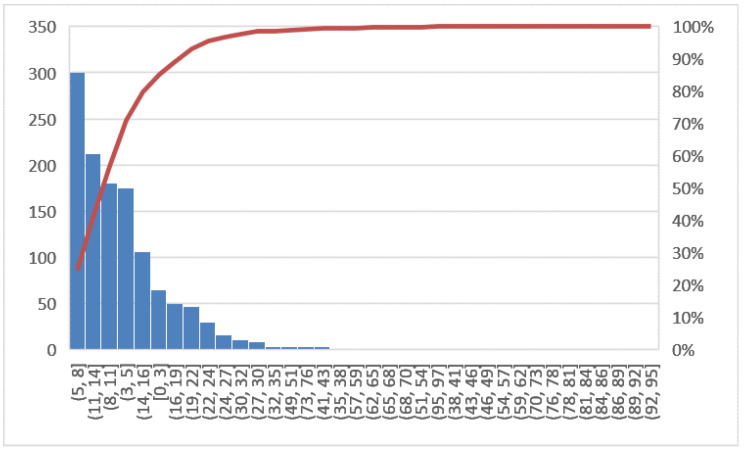
Pareto diagram of LOS (2020, 2021).

**Figure 7:**
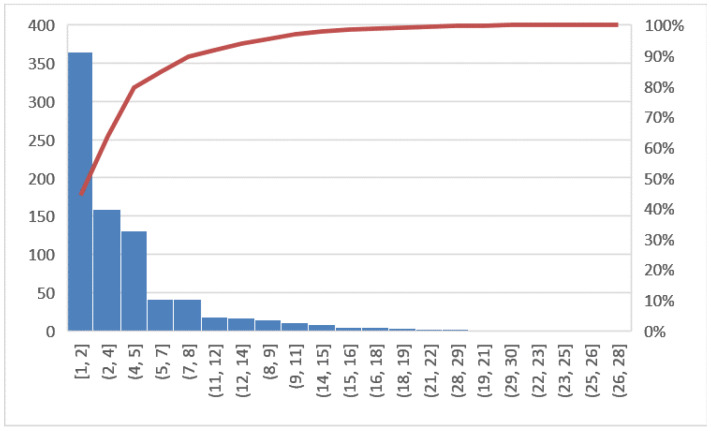
Pareto diagram of number of DH admission (2020, 2021).

**Figure 8:**
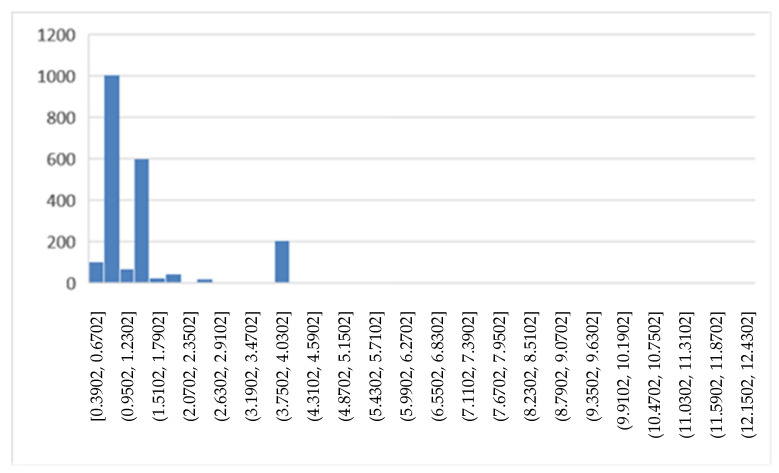
DRG relative weight (2020, 2021).

In this section, a comparison of before and after the COVID-19 emergency was implemented. First, the Pareto charts shown in Figure 6 and Figure 7 compare the LOS and Figure 8 the DRG relative weight in the two years before and after COVID-19, respectively.

**Figure 9:**
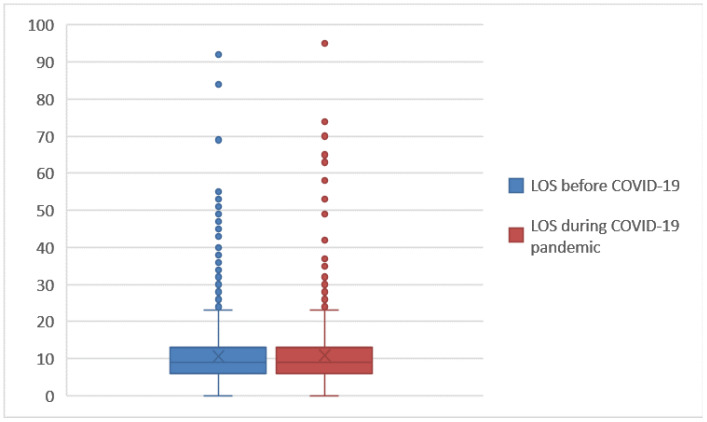
Box plot of LOS (2018–2019 vs. 2020–2021).

**Figure 10:**
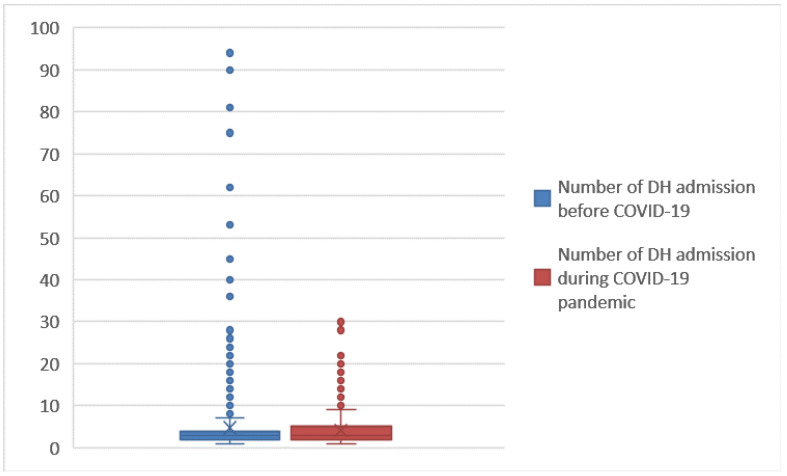
Box plot of number of DH admissions (2018–2019 vs. 2020–2021).

Comparing Figure 4 and Figure 7, we see how the values of the DRG relative weight increase in the period of the COVID-19 emergency. To facilitate the comparisons for LOS (Figure 9) and number of DH admissions (Figure 10), the following box diagrams have been created.

Figure 9 shows that there were no changes on the total LOS for hospitalized patients, while Figure 10 shows that a decrease in outliers occurred during the pandemic. Next, the logistic regression was implemented. Logistic regressions were used to test the association between the year of hospitalization (as a dependent variable) and the different risk factors under study (as explanatory variables): LOS, number of DH admissions, gender, age, DRG relative weight and mode of discharge. Logistic analysis has been carried out with IBM SPSS (Statistical Package for Social Science) ver. 27.3.1, and the results are reported in Table 5 and Table 6.

The first logistics analysis showed two significant variables—the DRG relative weight and LOS—while the second showed three significant variables—age, DRG relative weight and number of DH admissions. The descriptive statistics were performed using the chi-squared test and Kruskal–Wallis test, as appropriate. The level of significant α is equal to 0.05. The results are reported in Table 7.

Finally, with the statistical analysis, we obtained two other significant variables: DRG relative weight and the mode of discharge. The DRG relative weight has a *p*-value < 0.000 comparable to the mode of discharge, confirming the logistic regression.

**Figure 11:**
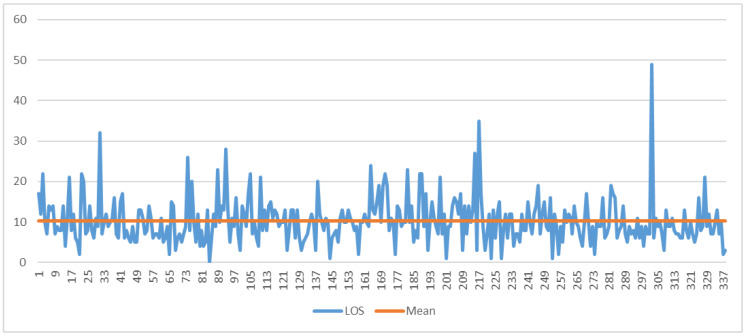
A run chart of total LOS for stroke patients during the COVID-19 pandemic. The orange line indicates the average value of 10.27 days.

In regard to stroke patients, Figure 11 shows the run chart obtained by analyzing the LOS of the 338 patients hospitalized in the years 2020–2021.

Comparing the results with those reported in Figure 4, it can be seen that the number of patients and the LOS decrease. To verify the veracity of this statement, statistical tests (Table 8) and logistic regression (Table 9) were implemented.

**Figure 12:**
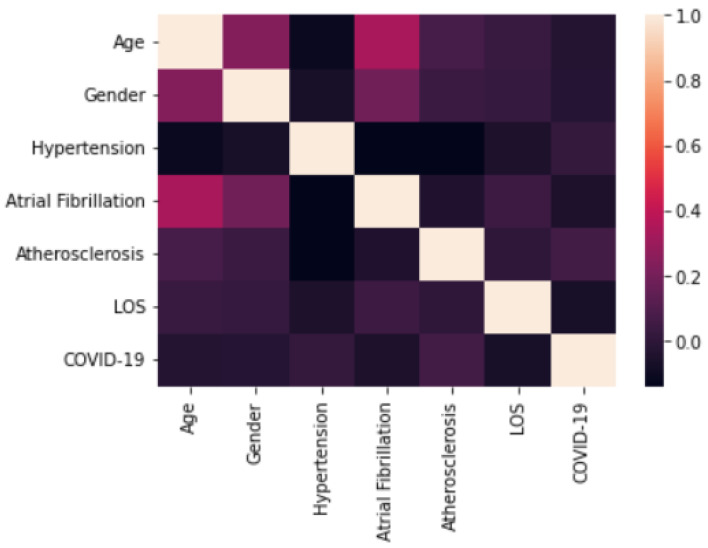
Correlation analysis.

The analysis of the results shows that there is a statistically significant difference in the total LOS. Finally, the ML algorithms were implemented. Figure 12 shows the analysis of the initial correlations.

As can be seen from Figure 12, the COVID-19 variable is only correlated with hypertension and atherosclerosis, with a coefficient of around 0.2.

### 3.2. Classification Results

In this section, different well-known machine learning models have been evaluated for classifying patients between the two identified classes. In particular, we designed a binary classification task on the basis of the features analyzed in Section 2.1 according to several measures (accuracy, precision, recall and F-measure) that were chosen for better handling the unbalanced problem, as shown in [38]. Each model is optimized on the basis of the training set to unveil the best parameters to deal with the designed task.

The outcomes of our analysis are summarized in Table 10. It is easy to note that SVM achieves the best performances in terms of accuracy (80% overall), precision and F-measure, being able to better handle this unbalanced dataset with respect to the other models. In fact, it is easy to see in Table 10 that SVM achieves a 10% to 28% F-measure score for the minority class with respect to the other models.

**Figure 13:**
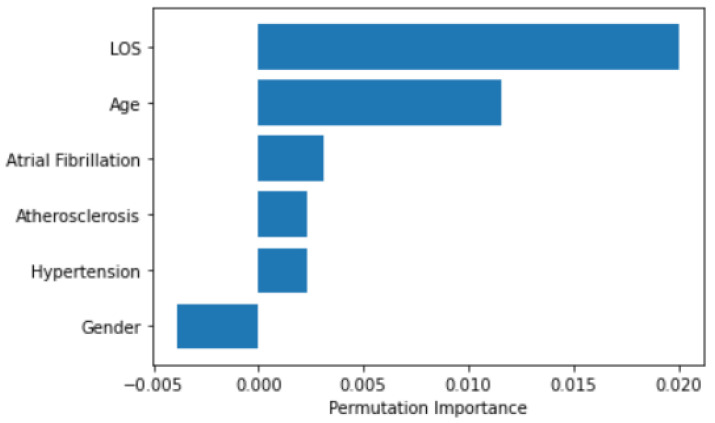
SVM permutation importance.

Table 11 shows the confusion matrix for this SVM model, where it is easy to note how the number of false classification samples is about the same for both classes. Furthermore, Figure 13 shows the importance of the permutation feature for the SVM model in order to establish which are the main features affecting the efficacy results.

By looking at the SVM coefficients, it is possible to identify the main features used in classification. In this case, the higher coefficient is associated with the LOS, in accordance with the results of the statistical analysis.

Finally, in Table 12, we show the hyperparameters identified for the SVM model during the training phase in order to improve the reproducibility of the analysis.

## 4. Discussion

In this study, the data relating to patients who had access to the COU of the Neurology and Stroke Unit of the San Giovanni di Dio e Ruggi d’Aragona University Hospital in the periods between 2018–2019 (pre-COVID-19) and 2020–2021 (during the COVID-19 emergency) were considered. The goal was to evaluate the impact of the COVID-19 pandemic on the ward’s health activities and to consider how the pandemic has affected the hospitalization of patients. To this end, we performed an analysis by comparing the 2018–2019 (pre-COVID-19) and 2020–2021 (during the COVID-19 pandemic) data; this was the data used to assess the impact of the pandemic. The following variables were analyzed for all patients: gender, age, LOS (length of stay), number of DH admissions, relative DRG weight and modality of discharge. Through the use of LSS methodology, with the implementation of the DMAIC cycle, it was possible to outline and compare the parameters characterizing the specific COU.

In particular, through the well-established DMAIC cycle, a rigorous definition of the problem to be addressed has been carried out during the define phase, where the process standards, the timing and, more importantly, the indicator to be measured as critical to quality (in this case, the LOS) have been clarified and agreed upon among all the healthcare staff involved in the reorganization of the COU. Afterwards, the measure and analyze phases helped in describing the so called “as is” process by collecting and analyzing data on the predefined indicators using both a Pareto chart and statistical analysis. In this phase, the statistical analysis supported the identification of the major characteristics of the dataset examined over the entire period under study, while the Pareto chart, followed by the run chart, helped in deepening the understanding and dynamics of the CTQ, i.e., the hospital stay, by outlining its evolution over time. The knowledge of the “as is” process and, more specifically, of the LOS acquired during the measurement phase of the DMAIC cycle allowed the project team to formalize the problem and hypothesize possible solutions for a better management of stroke patients during the COVID-19 pandemic. For this reason, national and regional guidelines and regulations were consulted, and decisions about strategies to improve patient management were proposed in compliance with the national and regional standards. In particular, a reorganization of the stroke network in the Campania region, based on the number of hospitalizations, has been taken into account as the major regional reference. Among the proposed strategies, improvement in the education and information of the healthcare staff about the COVID pandemic and related regulations has been suggested. Moreover, telestroke networks and preferred diagnostic-therapeutic assistance pathways have been set up in order to facilitate the management of stroke patients. The outputs of the final control phase of the DMAIC cycle, which have been extensively presented in the Results section of this work, reveal how the implementation of the proposed improvement measures have impacted the considered CTW and the related indicators examined in this case study. The main results highlight that the LOS plays a major role in the management of stroke patients (as demonstrated in the different regression and machine learning models implemented) and that a slight (although not very significant) decrease in the LOS has been achieved after the implementation of the improvement actions. Despite that, the results demonstrate a reduction in the number of DH admission. This is in accordance with other literature studies indicating that a reduction in hospital admissions for stroke patients is noted during the COID-19 pandemic [65,66]. In particular, the reduction effect is not as noticeable for the mean value as for the reduction in the outliers. Furthermore, the results demonstrated an increase in the value of the DRG relative weight; this indicates a higher complexity of the medical treatments carried out in 2020 and therefore, more appropriate hospital access. This is also in agreement with other studies [67] confirming that from both and economic and clinical perspective, the COVID-19 outbreak has paved the way for the development of novel strategies for a better management of stroke patients. Compared with our previous analysis, where only hospitalizations for the years 2019–2020 were studied, the growth of the relative weight DRG is confirmed. As for the stroke patients, both the statistical analysis and the logistic regression showed a significant reduction in LOS, and therefore, a greater turnover in the use of beds. The implementation of ML algorithms, with an accuracy of 80%, helped to more fully explain the substantial differences that exist between the two different groups of stroke patients, such as by allowing an automatic classification of cases with high performance. In summary, the main novelties of this works compared to the state of the art are:

- Although several analyses have been made in the last two years to analyze COVID-19 pandemic, the majority of these have focused on how the virus spreads and what factors most impact this spread. This analysis focuses on the patients in order to evaluate and improve the understanding of the impact of the COVID-19 pandemic on a cohort of 1538 subjects,
- It analyzes changes due to COVID-19 in terms of LOS (length of stay), mode of discharge and DRG (diagnosis related group) relative weight.
- It combines the use of both Lean Sigma Approach and predictive machine learning tools in order to deepened and strengthen the analysis of the proposed case study.

The COVID-19 emergency led to a profound restructuring—in a very short time—of the Italian hospital system. In times of pandemics, a national health system must guarantee the best possible services to patients with non-communicable diseases. In particular, these systems must maintain their ability to operate effectively, especially for those patients with acute conditions such as stroke and myocardial infarction, in which the applicable treatments are always time-dependent. The limit of this study is that only the whole activity of the said COU was considered, without focusing on the single case study.

## 5. Conclusions

In this study, the impact of COVID-19 on the activities of the COU of the Neurology and Stroke Unit of the San Giovanni di Dio e Ruggi d’Aragona University Hospital was analyzed. The novel objective of this work is to analyze, from a Lean Six Sigma perspective, the containment actions implemented by the hospital, in order to rigorously evaluate their impact on organizational, clinical and demographic variables of the neurological patients. In addition, a specific focus on stroke patients allowed us study a specific pathology in depth.

The results show an increase in the complexity of cases treated by the department and a reduction in LOS for patients who are admitted for stroke.

Future work will focus on a detailed analysis of the individual pathologies constituting the case mix of the department and a comparison with what has occurred in other departments of the hospital, as well as what has occurred in the same type of COU in other hospitals similar in territory and dimensions.

## Figures and Tables

**Table 1:** Project chart.

Project Title: Lean Six Sigma to Assess How COVID-19 Has Changed the Complex Operative Unit of the Neurology and Stroke Unit Patients’ Management: A Single Center Study.
Question: The inappropriate prolongation of hospital stays.
CTQ: LOS (Length of stay), mode of discharge and DRG relative weight.
Target: To realize corrective measures in order to reduce the CTQ.
Timeline: Define—January 2018–February 2018 Measure—March 2018–February 2020 Analyze—February 2020–8 March 2020 Improve—8 March 2020–December 2020 Control—31 December 2021

**Table 2:** SIPOC for the Neurological Clinic and Stroke Unit.

***Supplier*:** **Neurological Clinic and Stroke Unit of San Giovanni di Dio e Ruggi d’Aragona University Hospital**	***Input*:** **Hospital Services**	***Process*:** **Care Process** **(Administration** **Services)**	***Output*:** **Diagnostic and** **Therapeutic** **Information**	***Customers*:** **Patients at** **San Giovanni di Dio e Ruggi d’Aragona University Hospital**

**Table 3:** Study population features.

Features	2018 (N = 1239)	2019 (N = 1287)	2020 (N = 1148)	2021 (N = 896)
**Gender**				
**M**	627	655	612	462
**F**	612	632	536	434
**Age**				
**Age ≤ 50**	262	252	256	190
**50 < Age ≤ 70**	478	477	463	344
**Age > 70**	499	558	429	362
**Mode of discharge:**				
**Deceased**	39	60	47	48
**Ordinary at home**	1097	1115	1002	747
**Protected in non-hospital facilities**	1	-	1	2
**Home hospitalization**	-	-	-	-
**Voluntary**	56	44	33	22
**Transferred to another hospital**	6	7	21	20
**Transferred to another regime in the same institution**	10	12	14	21
**Transferred to rehabilitation institute**	24	48	28	32
**Protected with Integrated Home Assistance activation**	6	1	2	4
**DRG relative weight**				
**Mean**	1.10	1.20	1.27	1.44
Number of hospital admissions *				
**Mean**	5.38	4.28	3.80	4.42
Length of stay, LOS **				
**Mean**	10.53	10.83	10.67	11.18

* Only for 1610 patients with day hospital admissions. ** Only for 2971 hospitalized patients.

**Table 4:** New proposal for the COVID-19 emergency.

New Proposals for the Management of the COU of the Neurology and Stroke Unit during the COVID-19 Emergency	
Improve education of health professionals and the public during the pandemic	Stroke is an emergency, and treatment is available. The benefit of being treated for life-threatening disease far outweighs the risk of being infected with COVID-19. High-standard treatment is guaranteed during the pandemic. Stroke patients will be managed under a “protected stroke code” to avoid infection.
Set up telestroke networks	Implementing the existing telestroke networks and avoiding futile transports. Starting telestroke pathways from the patient’s home, employing territorial emergency doctors and nurses who carry out evaluations, so as to eliminate any contact with the disease and the possibility of contagion. Using telemedicine at an in-hospital level to evaluate patients who are suspected of having an acute neurological pathology in the COVID units.
Reorganize stroke pathways with a “protected stroke code”	Evaluating patients with unknown COVID-19 status under a “protected stroke code” with appropriate personal protective equipment.
Facilitating new stroke treatment options	

**Table 5:** Logistic regression—Hospitalized patients.

	Exp(B)	95% C.I. for EXP(B)		*p*-Value
		Lower	Upper	
**Gender, Male**	1.081	0.929	1.256	0.314
**Age**	0.999	0.995	1.004	0.773
**DRG relative weight**	1.437	1.323	1.562	**0.000**
**Length of stay (LOS)**	0.985	0.975	0.996	**0.006**
**Mode of discharge**				
**Deceased**	0.879	0.264	2.923	0.833
**Ordinary at home**	0.714	0.222	2.294	0.572
**Protected in non-hospital facilities**	4.913	0.371	64.994	0.227
**Home hospitalization**	0.588	0.174	1.986	0.393
**Voluntary**	3.004	0.800	11.281	0.103
**Transferred to another hospital**	9.290	1.440	59.931	**0.019**
**Transferred to another regime in the same institution**	0.740	0.219	2.498	0.628

**Table 6:** Logistic regression—Patients with DH admissions.

	Exp(B)	95% C.I. for EXP(B)		*p*-Value
		Lower	Upper	
**Gender, Male**	1.111	0.910	1.356	0.301
**Age**	0.992	0.986	0.998	**0.011**
**DRG relative weight**	5.369	2.679	10.763	**0.000**
**Number of DH admissions**	0.978	0.961	0.997	**0.020**

**Table 7:** Statistical analysis.

	Group 1 (2018–2019)		Group 2 (2020–2021)		*p*-Value
	Mean ± Dev. Std	Median	Mean ± Dev. Std	Median	
**Gender**	-	-	-	-	0.101
**Age**	64.03 ± 17.86	67.00	63.17 ± 17.47	66.00	0.169
Number of DH admissions *	4.72 ± 7.80	3.00	4.08 ± 3.72	3.00	0.640
Length of stay (LOS) **	10.68 ± 7.98	9.00	10.89 ± 8.30	9.00	0.251
**DRG relative weight**	1.15 ± 0.72	0.913	1.35 ± 1.02	0.910	**0.000**
**Mode of discharge**	-	-	-	-	**0.000**

* Only patients with day hospital admissions. ** Only hospitalized patients are considered.

**Table 8:** Statistics analysis—Stroke patients.

	Class 1 (2019) N = 650	Class 2 (2020) N = 338	*p*-Value
**Age**			
**Mean**	74.09	73.22	0.133
**Gender**			
**Male**			0.343
**Male**	339	187	
**Female**	311	151	
**Hypertension**			
**No**	518	264	0.560
**Yes**	132	74	
**Atrial Fibrillation**			
**No**	499	276	0.076
**Yes**	151	62	
**Atherosclerosis**			
**No**	566	279	0.055
**Yes**	84	59	
**LOS**			
**Mean**	11.19	10.27	**0.037**

**Table 9:** Logistic regression—Stroke patients.

	OR	95% CI	*p*-Value
**Gender, Male**	0.998	0.987–1.009	0.673
**Age**	1.085	0.824–1.428	0.563
**Hypertension (No)**	0.925	0.665–1.288	0.645
**Atrial Fibrillation (No)**	1.242	0.870–1.774	0.232
**Atherosclerosis**	0.691	0.477–1.000	0.050
**Length of stay (LOS)**	0.976	0.955–0.999	**0.037**

**Table 10:** Performance metrics of all selected models in the test set.

Performance Metrics	Class	DT	GBT	RF	SVM	LR
Accuracy	Overall	0.72	0.64	0.72	0.80	0.52
Precision	1	0.61	0.65	0.81	0.82	0.52
	2	0.84	0.63	0.67	0.78	0.52
Recall	1	0.79	0.59	0.57	0.76	0.50
	2	0.68	0.68	0.87	0.84	0.55
F-measure	1	0.69	0.62	0.67	0.79	0.51
	2	0.75	0.65	0.76	0.81	0.53

**Table 11:** SVM confusion matrix.

Class	1	2
**1**	99	31
**2**	21	109

**Table 12:** Hyperparameters for SVM.

Parameter	Value
**Kernel**	RBF
**C**	1
**Gamma**	1

## Data Availability

The datasets generated and/or analyzed during the current study are not publicly available for privacy reasons, but are available from the corresponding author on rea-sonable request.
